# Annuloplasty effect of transcatheter edge-to-edge repair with MitraClip system on multiple degenerative mitral regurgitations

**DOI:** 10.1093/ehjcr/ytad328

**Published:** 2023-07-24

**Authors:** Motoki Fukutomi, Takayuki Onishi, Haruka Sasaki, Tetsuya Tobaru

**Affiliations:** Department of Cardiology, Kawasaki Saiwai Hospital, 31-27 Omiyacho, Saiwai-ku, Kawasaki-shi, Kanagawa, 212-0014 Kawasaki, Japan; Department of Cardiology, Kawasaki Saiwai Hospital, 31-27 Omiyacho, Saiwai-ku, Kawasaki-shi, Kanagawa, 212-0014 Kawasaki, Japan; Department of Cardiology, Kawasaki Saiwai Hospital, 31-27 Omiyacho, Saiwai-ku, Kawasaki-shi, Kanagawa, 212-0014 Kawasaki, Japan; Department of Cardiovascular Medicine, Chiba University Graduate School of Medicine, Chiba, Japan; Department of Cardiology, Kawasaki Saiwai Hospital, 31-27 Omiyacho, Saiwai-ku, Kawasaki-shi, Kanagawa, 212-0014 Kawasaki, Japan

**Keywords:** MitraClip, Mitral annuloplasty effect, Degenerative mitral regurgitation

## Abstract

**Background:**

Previous reports have shown a mitral annuloplasty-like effect after MitraClip, i.e. a shortening of the anterior-posterior diameter of mitral valve annulus. However, the clinical benefit of this phenomenon is unclear.

**Case summary:**

An 87-year-old woman with multiple degenerative mitral regurgitation (MR) jets, including a central-medial jet and a lateral jet, underwent MitraClip procedure. After a single clip implantation, anterior-posterior diameter of mitral annulus was shortened and both MR jets were significantly reduced.

**Discussion:**

Even in the case of multiple MR jets, a single clip deployment with the MitraClip system may provide an acceptable MR reduction if the clip shows the mitral annuloplasty effect.

## Case description

**Figure 1:**
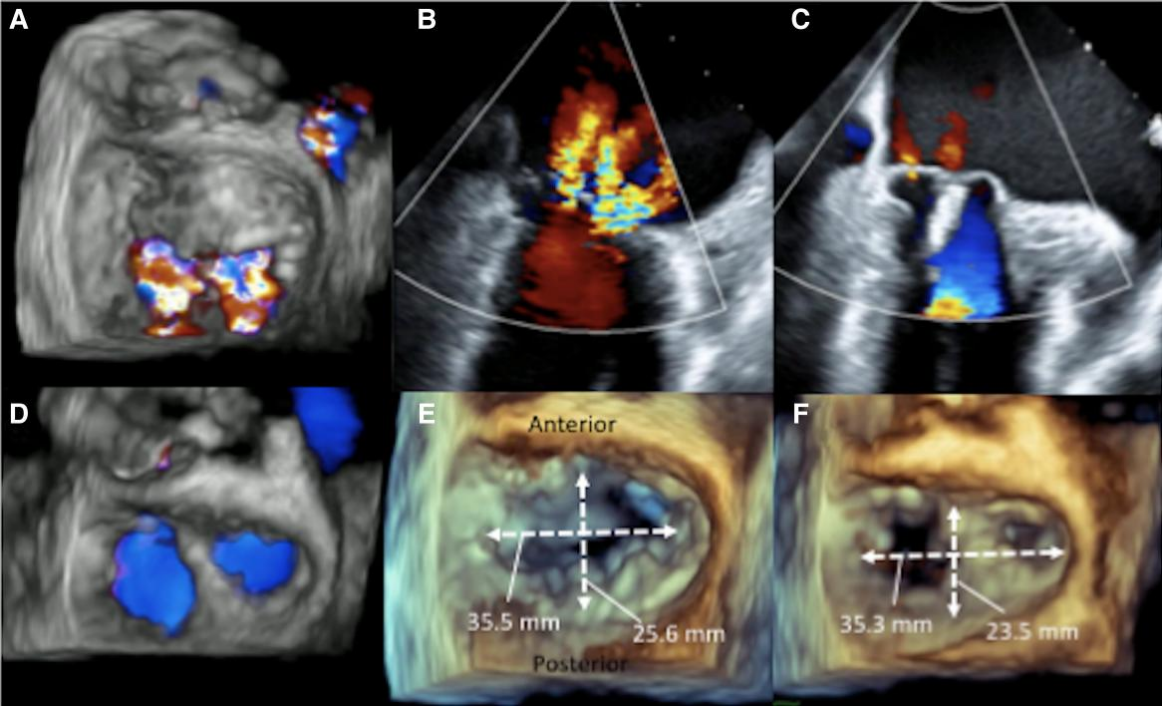
Transoesophageal echocardiography of the mitral annuloplasty effect after MitraClip implantation. (*A*) and (*B*) Two separated MR jets, including a central-medial jet and a lateral jet. (*C*) Significant reduction of all MR jets after a clip deployment. (*D*) NTW clip deployment in the 11–5 5 o’clock direction. (*E*) The mitral annular shape before MitraClip implantation. (*F*) Change of the mitral annular shape after MitraClip implantation with the reduction of the anterior–posterior diameter. MR, mitral regurgitation.

The patient was an 87-year-old woman with a history of repeated hospitalizations for heart failure with preserved ejection fraction with moderate to severe mitral regurgitation (MR) and persistent atrial fibrillation. MitraClip implantation was planned due to the patient’s frailty and high society of thoracic surgeons score of 20.65%. Transthoracic echocardiography (TTE) showed that the ejection fraction was 63% with a left ventricular end-diastolic diameter of 33 mm. Transoesophageal echocardiography (TEE) detected two separated degenerative MR jets with effective regurgitant orifice area of 0.34 cm^2^ (*Figure 1A and B* and Supplementary material online, *Video S1* and S*2*), including central-medial jet due to both A2 medial and P2 prolapse (see Supplementary material online, *Video S3*), and a lateral eccentric jet due to A1 prolapse (see Supplementary material online, *Video S4*). TEE measurements revealed that the mitral valve area was 3.2 cm^2^, the mitral annulus circumference was 108 mm, and the A2/P2 length was 21/10 mm.

Our first attempt was to grasp the medial side of A2/P2 using an NTW clip. To effectively reduce this MR jet while maintaining favourable leaflet coaptation, the clip was deployed adjusted to the 11–5 o’clock direction, perpendicular to the medial side of the mitral valve coaptation line. After the closure of the clip arms, the central-medial MR almost disappeared. At this time, the lateral MR jet was also substantially reduced. Neither the central-medial jet nor the lateral jet increased even after the release of the clip (*Figure 1C and D*, see Supplementary material online, *Video S5, S6* and S*7*). With a mean mitral gradient of 5 mmHg, the procedure was completed without a second clip implantation. Follow-up TTE at 3 months after MitraClip showed no significant increase in MR on either side of the clip.

The 3D TEE images of the mitral annulus shape before and after MitraClip were compared. As shown in *Figure 1E* and *F*, the mitral annular shape became more elliptical with the shortening of the anterior–posterior diameter after MitraClip, suggesting that this treatment provided a mitral annuloplasty-like effect. This effect may have resulted in a significant decrease of lateral MR due to a better coaptation of the A1/P1 and A2/P2 lateral. Several previous reports have already shown a mitral annuloplasty-like effect after MitraClip,^1^ but the clinical benefit of this phenomenon is unclear. This case clearly demonstrated that just a single clip reduced two separated MR jets with the annuloplasty effect. Even in the case of multiple MR jets, a single clip placement may provide acceptable MR reduction if the clip shows the mitral annuloplasty effect.

## Supplementary Material

ytad328_Supplementary_Data

## Data Availability

The data underlying this article will be shared on reasonable request to the corresponding author.

## Notes

**Consent:** The authors confirmed that informed written consent for the publication of this case report, including images and related text, was obtained from the patient in accordance with COPE guidelines.

**Funding:** None declared.
